# Sleep Problems and Drinking Frequency among Urban Multiracial and Monoracial Adolescents: Role of Discrimination Experiences and Negative Mood

**DOI:** 10.1007/s10964-020-01310-1

**Published:** 2020-08-29

**Authors:** Patricia A. Goodhines, Jessica M. Desalu, Michelle J. Zaso, Les A. Gellis, Aesoon Park

**Affiliations:** 1 Syracuse University; 2 Clinical and Research Institute on Addictions, University at Buffalo

**Keywords:** multiracial, discrimination, emotion dysregulation, sleep, alcohol, adolescence

## Abstract

Mounting evidence suggests that multiracial adolescents may be at greater risk than their monoracial peers for both sleep problems and alcohol use. However, mechanisms underlying these uniquely-heightened risky health behaviors among multiracial adolescents remain a gap in the literature. This cross-sectional study examined a risk pathway involving discrimination experiences and negative mood underlying racial disparities in concurrent sleep problems and drinking frequency. Students at an urban, socioeconomically-disadvantaged high school (*N*=414; grades 9–11, *M*_age_=16.00 [*SD*=1.08]; 57% female; 17% multiracial, 41% Black, 22% White, 18% Asian, 2% Other; 12% Hispanic/Latinx) completed a survey. Path analysis demonstrated that associations of multiracial status with sleep problems (insomnia symptom severity and insufficient weekday sleep duration), but not drinking frequencies (past-year drinking or past-2-week binge-drinking frequencies), were explained by discrimination experiences and, in turn, negative mood. In ancillary analysis excluding White students, the serial indirect risk pathway was significant for both insomnia symptom severity and past-year drinking frequency outcomes. Discrimination experiences and negative mood may function as intermediate factors contributing to racial disparities in adolescent sleep problems, although longitudinal replication is needed.

## Introduction

Multiracial individuals are one of the fastest growing populations in the United States (United States Census Bureau 2012); however, the health behaviors of multiracial individuals, especially adolescents, remain under-researched. Research among multiracial adolescents is important because findings from monoracial adolescents may not apply to adolescents with complex racial identities and distinct experiences (Shih and Sanchez 2009). Moreover, there is mounting evidence that multiracial adolescents may be at greater risk than their monoracial peers for health outcomes, including sleep problems (Udry et al. 2003) and alcohol use (Goings et al. 2018). Despite known associations of sleep problems and alcohol use by which both are reciprocally exacerbated over time (Koob and Colrain 2019), mechanisms underlying these heightened risk behaviors among multiracial adolescents remain a gap in the literature.

### Risk Mechanisms Underlying Sleep Problems and Alcohol Use among Adolescents

The *Integrative Temporal Model of Youth Sleep and Substance Problems* (Edwards et al. 2015) posits that exposure to stressors induces emotional dysregulation, which in turn increases risk for both sleep problems and risky substance use (including alcohol). Specifically, exposure to stressors induces emotion dysregulation, such as negative (depressed and/or anxious) mood. In turn, this negative mood is theorized to confer risk for both sleep and alcohol use in adolescents. Regarding alcohol behaviors, associations with stressors and negative mood have been well-documented (Keyes et al. 2012), although evidence for this association is relatively less researched in adolescents compared to adults. Regarding sleep problems, emerging research in adolescents has likewise documented associations with exposures to stressors (e.g., quiz task) and resulting negative mood (e.g., tonic arousal; Beebe et al. 2010).

#### Sleep and alcohol use among multiracial adolescents.

Up to 34% of adolescents endorse past-year complaints of insomnia symptoms (e.g., difficulties initiating and/or maintaining sleep lasting 2 weeks or longer; Blank et al. 2015) and 69% endorse insufficient sleep duration (Meldrum and Restivo 2014). In particular, multiracial adolescents may be at greater risk for sleep problems than monoracial peers (Udry et al., 2003). A nationally-representative study of U.S. adolescents (National Longitudinal Study of Adolescent Health [Add Health]) demonstrated that multiracial adolescents (7% of sample) endorsed greater sleep problems relative to monoracial peers (i.e., White, Black, Asian, and Other race; Udry et al., 2003). However, specific sleep patterns among multiracial adolescents remain under-studied in the literature, as these individuals are often combined into an “other” race (e.g., Hawkins and Takeuchi 2016) or general “minority” (e.g., Spilsbury et al. 2004) category, possibly due to small sample sizes of multiracial adolescents.

Parallel to sleep problems, adolescence is a critical period for the exacerbation of alcohol use. In 2017, 5.5 million U.S. adolescents ages 12–17 reported past-year drinking (Substance Abuse and Mental Health Services Administration 2018). In particular, multiracial adolescents may be at greater risk for alcohol use (Goings et al. 2018), compared to minority monoracial peers. A nationally-representative study of U.S. adolescents (National Survey on Drug Use and Health [NSDUH]) demonstrated that multiracial adolescents (4% of sample) were more likely to use alcohol compared to their minority monoracial peers (e.g., Black adolescents), but use less than White peers (Goings et al. 2018). Generally, underage drinking is associated with substantial consequences, including academic impairment, drinking and driving, unprotected sex, assault, injury, and death (National Institute on Alcohol Abuse Alcoholism [NIAAA] 2017).

#### Sleep and alcohol use as multifinal outcomes.

Although extensive literature has documented the reciprocal, feed-forward association of sleep problems and alcohol use among adults (Pasman et al. 2020), mechanisms in play at earlier stages of development remain to be fully elucidated. Indeed, adolescent sleep problems have been highlighted as a risk factor for downstream alcohol use (Wong et al. 2015), and adolescent alcohol use has been highlighted as a risk factor for downstream sleep problems (Hasler et al. 2014). However, integrated research of both sleep and alcohol use among adolescents is lacking relative to adult samples. Examination of the developmental stage of adolescence presents a unique opportunity to capture multifinal risk mechanisms underlying these two related health risk behaviors.

### Extending Theory of Risk Mechanisms to Multiracial Adolescents: The Role of Discrimination and Negative Mood

Discrimination may be an especially salient stressor for multiracial adolescents. Research demonstrates that multiracial individuals experience discrimination at comparable (Nadal et al. 2011) or even greater (Johnston and Nadal 2010) frequencies relative to their minority monoracial peers (e.g., Black adolescents). Discrimination is conceptualized as social adversity or unfair treatment based on membership to a group (e.g., race/ethnicity; Krieger 2001). Discrimination experiences have been associated with an array of adverse health outcomes in racially-diverse adolescent samples, including sleep problems (Yip et al. 2019) and substance use (e.g., alcohol; Fuller-Rowell et al. 2012). Regarding multiracial individuals specifically, although a limited literature has demonstrated associations of discrimination experiences with health consequences among multiracial adults (Charmaraman et al., 2014), comparable research remains lacking among the potential risk group of multiracial adolescents.

Evidence suggests that negative mood (i.e., stress response) may explain demonstrated associations of discrimination experiences with sleep problems and alcohol use. Consistent with theory, discrimination (i.e., stressor) has been associated with negative mood, including depression (Huynh and Fuligni 2010) and anxiety (i.e., stress response; Douglass et al. 2016). Further, negative mood has been highlighted as a risk factor for both adolescent sleep problems (Becker et al. 2017) and alcohol use (e.g., Schleider et al. 2019). Indeed, research has demonstrated the serial association of discrimination with mood dysregulation and in turn downstream health consequences, both among racial minority adolescents (Majeno et al. 2018) and adults (e.g., Black and African American adults; Goosby et al. 2018). Because of the comparable or potentially greater levels of exposure to discrimination among multiracial (compared to monoracial) adolescents, effects of these stress responses on sleep and drinking may be especially salient among multiracial adolescents.

#### Developmental implications.

Developmental differences of multiracial and monoracial populations may explain observed disparities in risky health behaviors. Multiracial youth may accumulate relatively greater exposures to stressors such as microaggressions from parents and/or extended family (Franco et al. 2018) and/or conflicted racial identity (e.g., Harris et al. 2017). In turn, ensuing stress responses while navigating a multifaceted identity in a monoracial-focused society (Jackson et al. 2012) may increase health risk across the lifespan. Thus, research of multiracial status is critical to explicate the potentially-novel risk mechanisms underlying the heightened risky health behaviors observed among multiracial adolescents. It is reasoned that investigation of multiracial (compared to monoracial) risk mechanisms may set the stage for more nuanced analysis of racially-specific developmental trajectories (including within-group variability) moving forward.

### Literature Gaps

Three important gaps exist in the adolescent sleep and alcohol literature. First, better understanding of risk pathways underlying the escalation of sleep problems and alcohol use in multiracial adolescents is needed given evidence of sleep-related disparity affecting racial minority adolescents overall and alcohol-related disparity affecting multiracial adolescents specifically. Most adolescent research has neglected to examine multiracial adolescents as a distinct group, limiting knowledge about this group’s unique risks. Second, findings from limited previous studies examining multiracial (compared to monoracial) adolescent sleep (Udry et al. 2003) and alcohol use (Goings et al. 2018) are drawn from samples that are nationally-representative in terms of urbanicity and socioeconomic status, and thus generalizability to specific subgroups of urban and socioeconomically-disadvantaged multiracial adolescents remains uncertain. Third, although adolescent sleep problems and substance use theoretically share an emotion dysregulation risk pathway (Edwards et al. 2015), sleep problems and alcohol use have rarely been considered as multifinal outcomes with a common underlying mechanism.

## Current Study

Common risk mechanisms underlying diverse adverse health outcomes experienced by multiracial (compared to monoracial) individuals remain a critical gap in the literature. This study investigated theorized associations of race with sleep problems and alcohol use (Edwards et al. 2015) among multiracial and monoracial adolescents from an urban and socioeconomically disadvantaged high school using cross-sectional data. Previous research reviewed herein demonstrating associations of race with discrimination (Johnston and Nadal 2010), discrimination with negative mood (e.g., Douglass et al. 2016), and negative mood with both sleep problems (Becker et al. 2017) and alcohol use (e.g., Schleider et al. 2019) among adolescents further support the theoretical and analytical model. Thus, it was hypothesized that the association of multiracial (compared to monoracial) status with sleep problems and drinking frequency would be explained by discrimination experiences and in turn negative mood. Ancillary analysis also investigated the same mechanism in a sample excluding White students to explore whether with the risk mechanism holds when comparing multiracial adolescents to minority monoracial groups only (i.e., Black, Asian, and Other race).

## Methods

### Participants and Procedure

Cross-sectional data were drawn from 414 adolescents (*M*_age_=16.00 years [*SD* =1.08], 57% female) at an urban public high school in the northeastern U.S. as part of an ongoing, longitudinal, genetically-informed alcohol study, *Project Teen*. The sample consisted of 17% multiracial and 83% monoracial students (41% Black or African American, 22% White, 18% Asian, 2% Native Hawaiian or Other Pacific Islander, and 1% American Indian or Alaska Native), and overall 12% of this sample identified as Hispanic/Latinx ethnicity (regardless of race; see Table 1). Students were eligible if they were English-speaking and currently enrolled in the 9th, 10th, or 11th grade at the time of study enrollment; students were ineligible if they were in the 12^th^ grade at the time of enrollment, because they were expected to graduate from high school prior to the one-year follow-up assessment for the larger study and thus their sleep and alcohol behaviors may differ from those of high school students. This sample represents substantial socioeconomic disadvantage on average, as indicated by 87% eligibility for a free or reduced-price lunch program, 20% having a primary care giver without a high school diploma, and 75% living in a neighborhood where at least 20% of residents fall below the federal poverty line (United States Census Bureau 2017). Regarding average family structure, 14% of this sample reported a single primary caregiver and 93% endorsed having siblings. The current sample is representative of high school students in this district in terms of race, ethnicity, and socioeconomic status, although the proportion of girls in this sample is higher than that of the school district (consistent with other health survey studies; e.g., Geers et al., 2017).

All study procedures were approved by the Institutional Review Board and the school district. A Certificate of Confidentiality was obtained from National Institutes of Health to protect confidentiality of sensitive information (e.g., illicit substance use). Participants were recruited through class visits. Informed assent and guardian consent was obtained from all participants enrolled in the study. Participants received an email link to two web-based surveys assessing diverse health behaviors (including sleep problems and drinking frequency). Most participants completed the survey during a regular class period on an individual computer fitted with a privacy screen in the school computer lab (with no teachers present), but a few participants completed the survey via a personal phone/computer after school hours. Throughout survey completion, students were reminded by both electronic prompts and research staff that their answers were confidential. Students were compensated with up to $15 via gift card for completing the survey; additionally, any student who returned a completed parental consent (regardless of their actual participation or their guardian’s consent to participate) could receive extra credit based on individual teacher discretion. All Year 1 participants have been invited for the Year 2 survey for the larger longitudinal study, although the current analyses use cross-sectional data from Year 1. Students were also invited to provide cheek swabs for genotyping for an additional $5 as part of the larger study, although this was not required to participate in the survey study.

### Measures

#### Sleep problems.

The 7-item Insomnia Severity Index (Bastien et al. 2001) assessed current (i.e., last two weeks) severity of insomnia symptoms (e.g., difficulty falling asleep, difficulty staying asleep, diurnal functional impairment due to sleep problems). Responses were based on a 5-point Likert scale ranging from 0 to 4, with anchors relevant to the item assessed and higher values indicating more severe symptoms (e.g., *none* to *very severe*). A sum score (*α*=.76) was used for analyses; sum scores 0–7 are interpreted as no clinically significant insomnia, 8–14 as sub-threshold insomnia, 15–21 as clinical insomnia of moderate severity, and 22–28 as severe clinical insomnia (Buysse et al. 2006; Smith and Wegener 2003). Insomnia symptom severity was chosen for main analysis, which is well-validated and standard in the sleep literature, to maximize reliability of insomnia symptom assessment (see Buysse et al. 2006). This measure is commonly utilized in adolescent sleep studies (e,g., Zhang et al. 2016). Further, current sum scale scores are preferred to alternative dichotomized sleep outcomes (e.g., insufficient sleep duration, below) due to their superior ability to capture nuanced between-person variability.

In ancillary analysis, past-2-week insufficient (<7 hours) weekday and weekend sleep duration was used as a secondary sleep outcome to further explore sleep problems commonly endorsed during adolescence (e.g., Meldrum and Restivo 2014). Two items assessed subjective average sleep duration on weekdays and weekends (i.e., “What was your average total sleep time per day during the [weekdays/weekends] for the past two weeks?”) with responses based on a 7-point Likert scale ranging from 0 (*0–5 hours*) to 6 (*Greater than 10 hours*). Responses to these two variables were coded dichotomously (0=*meets recommendations for sleep duration [7 or more hours]*, 1=i*nsufficient sleep duration [0–7 hours]*), consistent with sleep duration recommendations for this age group (Hirshkowitz et al. 2015) and previous studies of adolescent sleep (e.g., Wheaton et al. 2016).

#### Alcohol use.

In main analysis, one item assessing past-year drinking frequency was used as the main alcohol outcome given its superior performance as a screener for adolescent alcohol problems (as compared to quantity-based measures; Chung et al. 2012). Participants responded to this item (i.e., “On how many occasions have you had alcoholic beverages to drink [more than just a few sips] during the last 12 months”; Johnston et al. 2016) based on a 7-point Likert scale ranging from 0 (*Never*) to 6 (*40 or more occasions*). The past-year assessment timeframe (as opposed to shorter timeframes such as past-2-week) was chosen for main analysis due to the low prevalence and infrequent and opportunistic nature of adolescent drinking (Windle 2016). A shorter timeframe may compromise reliability and in turn validity because highly infrequent behaviors captured within a short term period are likely to be determined by random chance (such as accidental availability) instead of stable drinking patterns.

In ancillary analysis, one item assessing past-2-week binge drinking frequency was used as a secondary alcohol outcome to examine high-risk drinking behavior in adolescents (NIAAA 2017). Participants responded to this item (i.e., “Think back over the last two weeks. How many times have you had [male: 5; female: 4] or more drinks in a row?”; Johnston et al. 2016). Response options were based on a 6-point Likert scale (0=*non*e, 1=*once*, 2=*twice*, 3=*3–5 times*, 4=*6–9 times*, 5=*10 or more times*). Despite a low prevalence of the past 2-week binge drinking in our sample (*n*=140 did not binge drink), the original Likert-scale scores were used to retain potentially-important variability in past-2-week binge drinking frequency among lifetime drinkers (*M*=0.30 [*SD*=0.89], median=0, skew=3.85; *n*=15 reported 1 occasion, *n*=6 reported two occasions, *n*=0 reported 3–5 occasions, *n*=2 reported 6–9 occasions, and *n*=3 reported 10 or more occasions). Notably, ancillary path analyses with a dichotomized binge drinker status outcome (0=*No*, 1=*Yes*) yielded consistent patterns of significance for serial indirect effects.

Participants were provided with the definition of a drink as a 12-oz. can or bottle of beer, a 5-oz. glass of wine, 8-oz. glass of malt liquor, or 1.5 oz. of hard liquor straight or in a mixed drink, along with pictures of standard drinks. Lifetime alcohol abstainers (*n*=235; 57% of sample) were coded as missing on drinking frequency variables (i.e., lifetime abstainers were not asked about alcohol consumption). Notably, results of the main path analysis are consistent when lifetime alcohol abstainers are alternatively instead coded as ‘0’ (indicating their past-year drinking frequency is a zero).

#### Discrimination experiences.

The 9-item Everyday Discrimination Scale (Williams et al. 1997) assessed frequency of discriminatory experiences in day-to-day life (e.g., receiving poorer service than others in restaurants or stores, being called names or insulted). Response options were based on a 5-point Likert scale ranging from 0 (*Never*) to 4 (*Always*). A sum score was used for analyses; high internal consistency (*α*=.90) indicates that diverse discrimination experiences tend to cluster together. This measure has been used to assess experiences of discrimination among racially-diverse high school student samples (e.g., Majeno et al. 2018).

#### Negative mood.

The 4-item Patient Health Questionnaire (Kroenke et al. 2009) assessed past-2-week depression/anxiety symptom frequency. Responses were on a 4-point Likert scale ranging from 0 (*not at all*) to 3 (*nearly every day*). A sum score (*α*=.86) was used for analyses.

#### Race and racial/ethnic identity.

Self-identified race was assessed via a single item (i.e., “What race do you consider yourself to be? Race is a group of people who have similar and distinct physical characteristics.”; Centers for Disease Control and Prevention) with the following response options: Black or African American, American Indian or Alaska Native, Asian, Native Hawaiian or Other Pacific Islander, White, and Multiracial. Students who selected “multiracial” were presented with a subsequent investigator-developed item (i.e., “So you are multi-racial. What races do you consider yourself to be? Please select ALL that apply to you.”) with the same response options. For descriptive analysis, American Indian or Alaska Native and Native Hawaiian or Other Pacific Islander groups were combined into an “Other” category due to small sample sizes. Main analysis was conducted with a binary race variable (i.e., 17% multiracial and 83% monoracial; *N* = 414). Ancillary analysis was conducted with a binary race variable of multiracial (*n* = 70; 17%) compared to minority monoracial (*n* = 255; 62%; Black, Asian, and Other race) students, excluding White students. Ancillary analysis was not conducted to compare multiracial (*n* = 70; 17%) and White (*n* = 89; 22%) adolescents due to the small sample size and thus concern about statistical power.

For descriptive purposes, the 6-item Multigroup Ethnic Identity Measure-revised (MEIM-R; Phinney & Ong, 2007) assessed individual identification with one’s racial/ethnic group (e.g., “I have a strong sense of belonging to my own ethnic group” and “I have often talked to other people in order to learn more about my ethnic group”). Responses were on a 5-point Likert scale ranging from 1 (*strongly disagree*) to 5 (*strongly agree*). Individual mean scores of complete scale items (possible range: 1–5; *α*=.92) were used for analysis, with higher scores indicating higher levels of racial/ethnic identification.

#### Covariates.

Age, sex assigned at birth (0=*female*, 1=*male*), and ethnicity (0=*not Hispanic/Latinx*, 1=*Hispanic/Latinx*) were assessed and included as covariates based on previous findings demonstrating these sociodemographic characteristics’ associations with sleep (Guglielmo et al. 2018) and drinking (Terry-McElrath and Patrick 2018) among adolescents. Notably, Hispanic/Latinx ethnicity was assessed separately from race (i.e., two-item approach; consistent with National Institutes of Health 2015; Office of Management and Budget 1997). Eligibility for free or reduced-price lunch (0=*no*, 1=*yes*) was used as a proxy for socioeconomic status, which has been shown to be comparable to more complex measures of socioeconomic disadvantage in adolescent populations (Nicholson et al., 2014), and additionally included as a covariate to control for known associations with adolescent discrimination and relevant health outcomes (e.g., Fuller-Rowell et al. 2018).

### Data Analytic Strategies

Descriptive statistics, bivariate correlations, and race group comparisons were computed using *SPSS* Version 23 (IBM Corp. 2016). Race group comparisons and post-hoc analyses were conducted using one-way ANOVA tests for continuous variables and non-parametric Kruskal-Wallis tests for dichotomous variables, with pairwise comparisons adjusted by Bonferroni correction for multiple tests. Path analysis was conducted using *Mplus* Version 8.1 (Muthén and Muthén 2018), which allowed for estimation of complex relationships among multiple variables in a single model simultaneously. Full information maximum likelihood estimation (Graham et al. 2003) was used to accommodate missing data, including systematic missing data for lifetime abstainers on the past-year drinking variable (*n*=235; 57% of sample) and other unsystematic missing data (*n*=14; 3%); *n*=167 (40% of the sample) had complete data on variables included in the main path model (see Table 2). After accounting for systematic missing data for lifetime abstainers on the past-year drinking variable, individuals with unsystematic missing data (*n*=14) did not significantly differ from peers with complete data (*n*=400) by any descriptive covariates at *p*<.05. Both drinking frequency variables, which represent counts of observed behavior despite ordinal Likert response options, were treated as count variables in path analyses to enable specification of non-normal distribution and accommodate predictable positive skew of adolescent responses. Past-year alcohol use frequency (*M*=1.34; variance=1.59, dispersion parameter=0.00; *p*=.75) was conservatively modeled with a negative binomial (versus Poisson) distribution due to its relatively less restrictive modeling assumptions (Gardner et al. 1995), despite its nonsignificant dispersion parameter. Monte Carlo integration was used to model the influence of continuous variables (i.e., discrimination and negative mood) on the count outcome variable (Muthén and Muthén 1998–2017, pp. 526–529). Residual error covariance between sleep problems and drinking frequencies was modeled by specifying a latent factor allowing for one dimension of integration (Muthén and Muthén 1998–2017, pp. 726–727). As effect size measures, standardized regression coefficients are presented for paths to continuous variables (i.e. discrimination experiences, negative mood, insomnia symptom severity) and incidence rate ratios (*IRR*s) are presented for paths to the count alcohol outcome variable.

**Figure 1.**
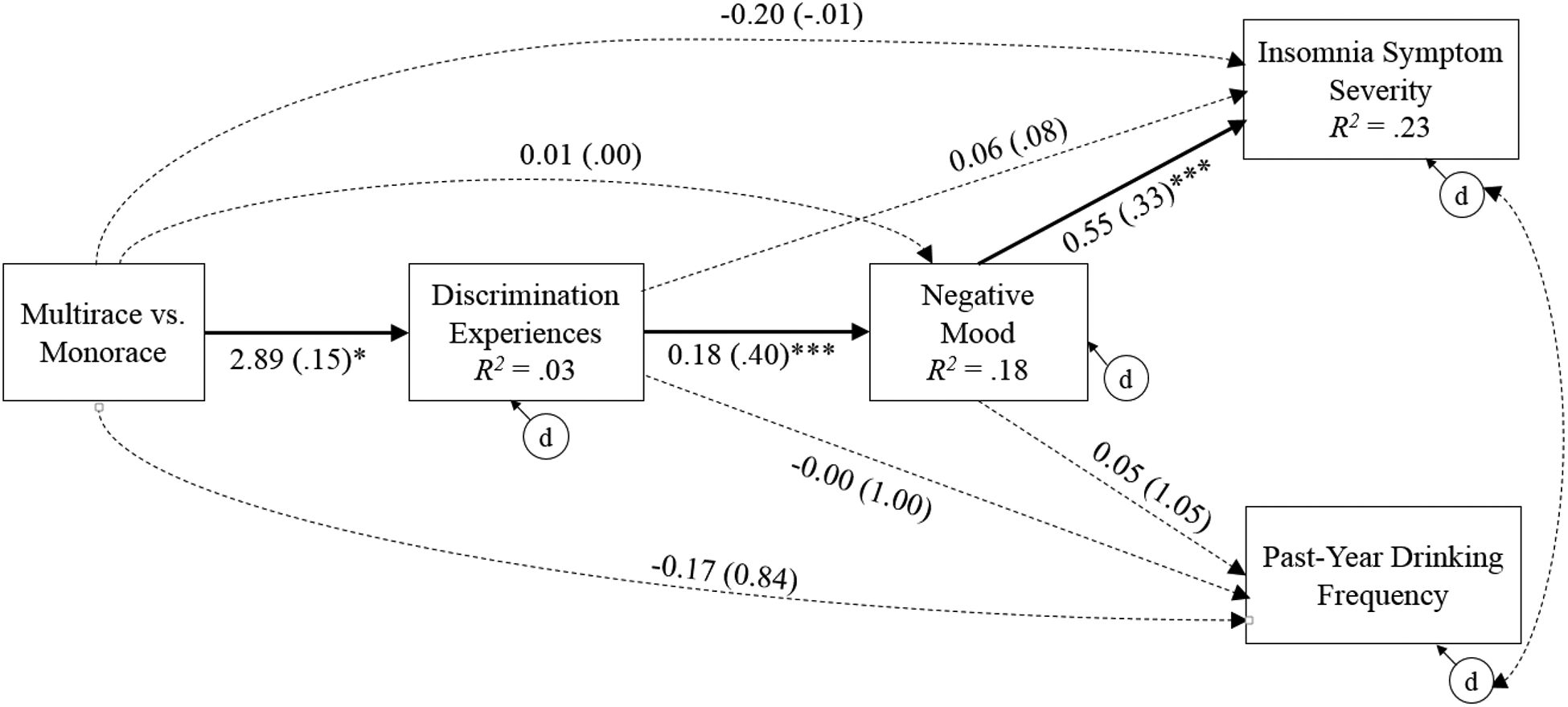
*N* = 414. Results from a fully saturated path model testing the serial indirect pathway for multiracial (vs. monoracial) adolescents after controlling for age, sex, Hispanic/Latinx ethnicity, and eligibility for free/reduced-price lunch; covariate pathways are not shown for simplicity. Unstandardized (and standardized) coefficients for paths leading to continuous variables (discrimination experiences, negative mood, and insomnia symptom severity) and unstandardized coefficients (and incidence rate ratios; *IRR*) for paths leading to a count variable (past-year alcohol frequency; negative binomial dispersion parameter=0.00, *p*=.75) are shown. Dotted paths indicate non-significant paths. The serial indirect pathway was significant for insomnia symptom severity (*b*=0.28, *SE* =.14, *p*=.05, 95% bootstrapped CI [0.04, 0.47]), but not past-year drinking frequency (*b*=0.02, *SE* =.02, *p*=.30, 95% bootstrapped CI [−0.00, 0.07]). d=disturbance/residual. *p<.05; ***p<.001.

For the main analysis, a fully saturated path model was estimated to test the serial indirect role of discrimination experiences (M_1_) and negative mood (M_2_) in the relationship of multiracial (compared to all monoracial groups) status (X) with two outcomes (that is, insomnia symptom severity [Y_1_] and past-year alcohol frequency [Y_2_]). Specifically, four indirect paths included multiracial status to discrimination experiences (*a* path of X → M_1_), discrimination experiences to negative mood (*d* path of M_1_ → M_2_), negative mood to insomnia severity (*b*_1_ path of M_2_ → Y_1_), and negative mood to the past-year alcohol frequency (*b*_2_ path of M_2_ → Y_2_). Thus, two indirect effects were calculated by multiplying unstandardized coefficients of the three indirect paths (Stride et al. 2015) leading to insomnia symptom severity (*a* * *d* * *b*_1_) and to binge drinking frequency (*a* * *d* * *b*_2_). Significance of serial indirect pathways were assessed using 95% confidence intervals (CIs) based on 10,000 resamples; CIs that did not encompass zero indicated significance. This bootstrapping method has demonstrated superior performance and power to detect indirect effects comparable to other traditional methods (e.g., Sobel test; Preacher and Selig 2012). Direct paths included a path from multiracial status to insomnia symptom severity (*c*_1_’ path of X → Y_1_) and a path from multiracial status to past-year alcohol frequency (*c*_2_’ path of X → Y_2_), after accounting for aforementioned indirect paths. Although not part of the hypothesized serial risk pathway, paths from multiracial status to negative mood (X → M_2_), discrimination experiences to insomnia symptom severity (M_1_ → Y_1_), and discrimination experiences to binge drinking frequency (M_1_ → Y_2_) were also estimated to avoid potential bias due to omitted paths. Participant age, sex, Hispanic/Latinx ethnicity, and eligibility free/reduced price lunch (as a proxy for socioeconomic status) were included as covariates (coefficients not shown in Figure 1 for simplicity).

Three ancillary analyses were conducted. First, to explore potential differences among racial minority groups (excluding White students), the main path analysis was replicated with an alternative racial predictor comparing multiracial and minority monoracial adolescents. Second, to test replicability of the hypothesized risk pathway to insufficient sleep duration (rather than insomnia symptom severity) and proximal risky drinking behavior (rather than alcohol consumption), the main path analysis was replicated twice with alternative sleep outcomes of dichotomized weekday and weekend insufficient (versus recommended) sleep duration, each modeled with the alternative alcohol outcome of past-2-week binge drinking frequency (*M*=0.30, variance=0.79). Third, another fully-saturated path model was conducted to explore the alternative hypothesis that the association of multiracial (compared to monoracial; X) status with negative mood (Y) was explained by discrimination experiences (M_1_) and, in turn, sleep problems and drinking frequency (modeled contemporaneously by specifying a latent factor allowing for one dimension of integration; M_2_). Consistent with the main analysis, age, sex, Hispanic/Latinx ethnicity, and eligibility free/reduced price lunch were included as covariates in all ancillary analyses.

## Results

### Descriptive Statistics

Race combinations for the full sample are presented in Table 1. Available data, means, standard deviations, and bivariate correlations are presented in Table 2. Among the full sample, 50% reported insufficient weekday sleep duration and 33% reported insufficient weekend sleep duration. On average, students reported sub-threshold insomnia symptom severity (*M*=8.18; *SD*=5.14) and drinking 1–2 occasions in the past year (*M*=1.34; *SD=*1.26; Median and Mode=1). Multiracial (compared to monoracial) status was significantly and positively associated with discrimination experiences (*r*=.14, *p*=.004). Further, discrimination experiences and negative mood were both positively correlated with insomnia symptom severity (*rs* = .22 to .38, *ps*<.001) and past-2-week insufficient weekday sleep duration (*rs* = .10 to .14, *ps*=.004–.04), but not past-2-week insufficient weekend sleep duration (*rs* = −.00 to .09, *ps*=.09–.98), past-year drinking frequency (*rs* = −.03 to .13, *ps*=.09–.75) or past-2-week binge drinking frequency (*rs* = .01 to .06, *ps*=.48–.93). Notably, students’ identification with their race/ethnicity was neutral on average (*M*=3.31 [*SD*=1.10]) and weakly negatively correlated with insomnia symptom severity (*r*=−.12, *p*=.02), but not with multiracial status, discrimination, negative mood, or sleep or alcohol outcomes (*p*s=.05–.84).

Means and standard deviations of all study variables by race, as well as race group comparisons, are presented in Table 3. Regarding race group comparisons of key variables included in main analyses, post hoc pairwise comparisons indicate that: (a) frequency of discrimination experiences was greater among Multiracial students (*M*=11.97 [*SD*=7.58]) compared to White students (*M*=8.60 [*SD*=6.19]); (b) negative good was greater among White students (*M*=3.75 [*SD*=3.22]) compared to Black students (*M*=2.54 [*SD*=3.04]); and (c) past-year drinking frequency was greater among White students (*M*=1.77 [*SD*=1.37]) compared to Asian students (*M*=0.91 *SD*=[1.11]). No other significant race group differences were observed on key variables included in main analyses (*p*s>.05).

### Path Analysis

Results of the main path analysis are depicted in Figure 1. Consistent with hypotheses, a significant serial indirect path was found from multiracial (compared to monoracial) status to discrimination experiences, negative mood, and insomnia symptom severity in sequence (*b*=0.28, *SE*=.14, *p*=.05, 95% bootstrapped CI [0.04, 0.47]) after controlling for covariates. Specifically, multiracial status was positively associated with discrimination experiences (*b*=2.89, *SE*=1.16, *β*=.15, *p*=.01), which was positively associated with negative mood (*b*=0.18, *SE*=0.04, *β*=.40, *p*<.001), which in turn was positively associated with insomnia symptom severity (*b*=0.55, *SE*=0.07, *β*=.33, *p*<.001). After accounting for indirect paths and covariates, multiracial status was not directly associated with insomnia symptom severity (*b*=−0.20, *SE*=0.67, *β*=−.01, *p*=.77), indicating that the association of multiracial status with insomnia symptoms severity was fully explained by discrimination experiences and negative mood. Inconsistent with hypotheses, negative mood was not associated with past-year drinking frequency (*b*=0.05, *SE*=0.04, *IRR*=1.05, *p*=.21) after controlling for covariates. Thus, the association of multiracial status with past-year drinking frequency was not serially explained by discrimination experiences and negative mood (*b*=0.02, *SE* =.02, *p*=.30, 95% bootstrapped CI [−0.00, 0.07]). Further, multiracial status was not directly associated with past-year drinking frequency (*b*=−0.17, *SE*=0.33, *IRR*=0.84, *p*=.60) after accounting for indirect paths and covariates.

### Ancillary Analyses

First, a comparison of multiracial to all minority monoracial (including Asian, Black, and Other adolescents, but excluding White adolescents) students was used as a predictor instead of a comparison of multiracial group with monoracial groups (including Asian, Black, “Other,” and White adolescents). Results demonstrate a significant serial indirect pathway to both insomnia severity (*b*=0.27, *SE*=0.10, *p*=.01, 95% bootstrapped CI [0.21, 0.55]) and past-year alcohol frequency (*b*=0.03, *SE*=0.01, *p*=.04, 95% bootstrapped CI [0.01, 0.05]).

Second, insufficient (<7 hours per night) sleep duration on weekdays and weekends were examined as sleep outcomes separately instead of insomnia symptom severity. Consistent with main analyses, results demonstrate a significant serial indirect pathway to insufficient *weekday* sleep duration (*OR*=1.05, *SE*=0.03, *p*=.13, 95% bootstrapped CI [1.01, 1.10]), but not past-2-week binge drinking (*b*=0.06, *SE*=0.05, *p*=.23, 95% bootstrapped CI [−0.00, 0.14]). Inconsistent with main analyses, results demonstrate a nonsignificant serial indirect pathway to insufficient *weekend* sleep duration (*OR*=1.01, *SE*=0.04, *p*=.90, 95% bootstrapped CI [0.94, 1.07]) and past-2-week binge drinking *b*=0.05, *SE*=0.04, *p*=.22, 95% bootstrapped CI [0.00, 0.12]). Notably, patterns of significance for serial indirect effects remained consistent after excluding students whose responses to respective sleep duration variables were greater than 10 hours (weekdays: *n*=18 [4%]; weekends: *n*=36 [9%]) or missing (*n*=7; 2%).

Third, to test an alternative risk pathway, a path model examined whether the association of multiracial (compared to monoracial) status with negative mood was explained by discrimination experiences, and in turn sleep problems and drinking frequency (modeled contemporaneously). Results demonstrate a significant serial indirect pathway underlying associations of multiracial (compared to monoracial) status with negative mood involving discrimination experiences and in turn sleep problems (*b*=0.08, *SE*=0.05, *p*=.13, 95% bootstrapped CI [0.01, 0.19]), but not involving discrimination experiences and in turn drinking frequency (*b*=0.01, *SE*=0.01, *p*=.30, 95% bootstrapped CI [−0.01, 0.03]).

## Discussion

Despite mounting evidence for health disparities in sleep problems and alcohol use affecting multiracial (compared to monoracial) adolescents, risk mechanisms underlying these multifinal outcomes remain largely under-researched. This study investigated a racially-relevant risk pathway theoretically underlying the racial disparity in adolescent sleep problems and alcohol use (Edwards et al. 2015) in a racially-diverse sample of urban and socioeconomically-disadvantaged adolescents. Overall, current findings indicate that discrimination experiences and in turn negative mood may function as intermediate factors contributing to racial disparities in adolescent sleep problems, although longitudinal replication is needed.

Consistent with hypotheses, results demonstrate an indirect risk pathway from multiracial status to sleep problems via discrimination experiences and negative mood. This finding adds to past research documenting positive associations of discrimination exposure with sleep dysregulation among minority monoracial individuals (e.g., Black adults; Petrov & Lichstein, 2016) by demonstrating that discrimination may be a uniquely salient risk factor underlying insomnia symptom severity and insufficient weekday (but not weekend) sleep duration among multiracial adolescents. Further, results are also in line with prior studies demonstrating associations of discrimination exposure with negative mood among minority monoracial groups (e.g., Black; Pieterse et al. 2012). Overall, these novel results add to emerging research suggesting that sleep problems in response to stress (Beebe et al. 2010) may be more salient among multiracial adolescents compared to majority (i.e., White) and minority monoracial (e.g., Asian and Black) adolescents. An alternative risk pathway is supported by ancillary analysis, such that the association of multiracial (compared to monoracial) status with negative mood was explained by discrimination experiences, and in turn sleep problems (but not drinking frequency). This alternative risk mechanism is likewise supported by previous findings, as known associations of discrimination experiences with negative mood (e.g., Douglass et al. 2016) may be explained by reactionary disruptions to health behaviors which precipitate negative mood in adolescence, such as sleep problems (e.g., Orchard et al. 2020). Longitudinal analysis is needed to resolve the complex reciprocal influences among discrimination, negative mood, and sleep that potentially contribute to exacerbation of symptoms over time (for a review of complex mechanisms potentially underlying reciprocal mood-sleep associations, see Blake et al. 2018).

Inconsistent with hypotheses and theorized associations of substance use (Edwards et al. 2015), results do *not* demonstrate an indirect risk pathway from multiracial status to past-year drinking frequency via discrimination experiences and negative mood. Negative mood may not be a salient risk factor for drinking during this developmental period, because drinking is more strongly associated with enhancement and social motives (versus coping with negative mood; Pompili and Laghi 2019) during adolescence. Thus, mid-adolescence may be premature for detection of this risk pathway to alcohol use behaviors, which may become relevant during emerging and young adulthood with greater prevalence of drinking (Delker et al. 2016) and coping-motivated drinking (Littlefield et al. 2010). It is noteworthy that the hypothesized risk mechanism was non-significant for drinking frequency outcomes regardless of measurement timeframe (i.e., past-year drinking or past-2-week binge drinking). However, results of ancillary analysis examining multiracial (compared to minority monoracial) status (i.e., excluding White students) support a significant indirect pathway from past-year drinking frequency via discrimination experiences and negative mood. These ancillary results are consistent with past research highlighting multiracial adolescents as a risk group for alcohol use compared to minority monoroacial peers (e.g., Black peers), but not White peers (Goings et al. 2018). Future research is needed to replicate analyses in larger samples with more variability in drinking behavior during late adolescence and adulthood to further elucidate this potential risk mechanism underlying multiracial adolescent drinking.

Results should notably be interpreted considering key sample characteristics. First, because socioeconomic status has been negatively associated with adolescent sleep problems (Gellis, 2011) and alcohol use (Miech, 2020), a lower prevalence of these risky health behaviors may be expected among samples that are higher socioeconomic status. Second, because high-risk urban settings characterized by neighborhood disorder (e.g., high population density, physical deterioration, and rates of crime and drug use; Arthur et al. 2002) and disadvantage (e.g., poverty, prejudice, and unfair treatment; Wilson 1987) may increase risk for adolescent substance use (including alcohol; e.g., Mason et al. 2017), it may be expected that socially-determined risk mechanisms underlying adolescent alcohol use may differ across samples of heterogenous urbanicity and neighborhood context. Overall, current results may be unique to the current urban, racially-diverse, and low-socioeconomic status sample. As such, generalizability of current preliminary findings regarding this theorized multifinal risk pathway to sleep problems and alcohol use among multiracial youth remains unknown and replication is needed among diverse demographic adolescent samples.

The current findings may also have important developmental implications for multiracial risky health behaviors over time. Prospective associations between sleep and alcohol use are known to be bi-directional (Haario et al. 2013), engaged in a feed-forward cycle by which each is exacerbated over time (Koob and Colrain 2019). Indeed, adolescent sleep problems have demonstrated longitudinal associations with increased alcohol problems (e.g., Wong et al. 2015), and alcohol has demonstrated an acute adverse impact on sleep among older adolescents (i.e., ages 18–21; Chan et al. 2013). Thus, the identification of a common risk pathway during mid-adolescence may help tailor prevention and intervention for the risk-group of multiracial adolescents to mitigate development of predictable down-stream consequences.

### Implications for Prevention and Intervention

Findings may inform prevention and intervention programming to curtail the sequela of discrimination experiences among multiracial adolescents. First, explication of a risk mechanism contributing to an emerging feed-forward risk process by which sleep problems and alcohol use are reciprocally exacerbated over time may assist in the early identification of at-risk youth and thereby the development of targeted prevention programming. Individuals who experience discrimination may implement coping strategies (e.g., rumination; Wang and Yip 2019) which exacerbate negative mood and increase downstream psychosocial and health consequences. Thus, intervention may validate individual experiences and offer emotion regulation techniques to aid with the emotional sequelae of perceived discrimination. Intervention may also seek to assist development and integration of complex multiracial identity, which has demonstrated a buffering role against the negative psychological effects of racial discrimination among multiracial adults (Jackson et al. 2012), but may be complicated by nuanced and/or conflicting racial identities (e.g., Harris et al. 2017). It is also important to consider individual experiences and responses to discrimination given that multiracial people are a heterogeneous group with variable risk and psychosocial protections (Goings et al. 2018). Specific to sleep problems, healthy sleep has demonstrated a protective role against the adverse sequela of discrimination in adolescents (Wang and Yip 2019). Evidence-based sleep interventions for adolescents include cognitive-behavioral therapy for insomnia (CBT-I; Dewald-Kaufmann et al. 2019), which might expand upon existing alcohol-specific content (consistent with recommendations for college students from Fucito et al., 2015). For example, limiting alcohol use before bed is recommended as a standard component of sleep hygiene education integrated into CBT-I (Stepanski & Wyatt, 2003). Integrated interventions may also include personalized feedback on drinking, drinking recommendations, and drinking reduction strategies (Fucito et al., 2015). Further, sleep interventions may explore culturally-informed sleep-inhibiting beliefs. Although sleep-related beliefs potentially novel to multiracial adolescents remain to be explicated, emerging research indeed highlights racial differences in beliefs about sleep (e.g., Black women endorsed less motivation to make time for sleep, as well as a stronger belief that sleepiness is due to laziness, compared to White peers; Grandner et al., 2013).

Systems-level interventions should also be considered to reduce frequency of the inciting stressor (i.e., discrimination), and therefore downstream health problems. Federal and local policies may discourage discrimination in schools by prioritizing a diverse teacher workforce and culturally-competent classroom management training for teachers (Trent et al. 2019). Further, family interventions for multiracial adolescents may seek to increase psychosocial protections such as parental engagement and substance use disapproval (Goings et al. 2018). Increased parental racial socialization (i.e., preparing multiracial children to face predictable discriminatory experiences) may further mitigate the effects of microaggressions toward multiracial people from parents and/or extended family (Franco et al. 2018).

### Limitations and Future Directions

Current findings should be interpreted within the context of some limitations. First, although the analytic model is in line with the theorized risk pathway to adolescent sleep problems (Edwards et al. 2015), it is not possible to infer causal relationships among study variables due to the cross-sectional nature of this study and the differing assessment time frames of key study variables. Second, self-report measures may be vulnerable to memory error and/or social desirability bias. Third, this study enrolled school-attending adolescents in a northeastern U.S. city with a high prevalence of socioeconomic disadvantage; thus, results might not generalize to non-school-attending adolescents or those from other geographical locations with different socioeconomic compositions. Fourth, this study assessed frequency (versus type) of general (versus specifically racial) discrimination, precluding more nuanced evaluation of how specific types of racial discrimination may uniquely explain disparate sleep and alcohol problems for multiracial adolescents. Fifth, current results of insomnia symptom severity may only generalize to insomnia (versus other sleep problems and experiences). That is, the Insomnia Severity Index (Bastien et al., 2001) is a useful tool for assessing insomnia symptoms (Buysse et al. 2006) and maps onto diagnostic criteria for insomnia disorder (American Psychiatric Association 2013; World Health Organization 1993), and as such may not generalize to other sleep disorders common to adolescence such as circadian phase disorders. Lastly, multiracial and monoracial students are not homogenous groups, and group-specific results might not generalize accurately to all group members; thus, additional research is needed to investigate within-group variability in this risk pathway.

Results of this study may inform future research. First, future studies should additionally explore group differences and associated risk conferred by type and stressfulness of discrimination experiences. Second, longitudinal research is needed to (a) replicate cross-sectional relationships over time to confirm temporal sequencing and (b) explore alternative and/or more comprehensive risk pathways underlying prospective sleep-alcohol associations among multiracial adolescents. Indeed, previous researchers have posited that multiracial adolescents may experience distinct pathways to substance use (including alcohol; Clark et al. 2013). For example, racial identification has demonstrated a protective role against drinking among Black adolescents (Stock et al. 2013); in contrast, multiracial adolescents’ racial identity development may be complicated by nuanced and/or conflicting racial identities (e.g., Harris et al. 2017), implicating greater susceptibility to alcohol use. Thus, future research is needed to explore the role of racial identity specifically in the current multifinal risk pathway to concurrent sleep problems and alcohol use. Third, future research of health behavior among multiracial adolescents should assess Latinx identification as a race category (i.e., one-question approach), consistent with literature recommendations for research with multiracial individuals (Charmaraman et al. 2014; Umaña-Taylor et al. 2014). Fourth, replication is necessary among both general adolescent samples and specific sub-samples of heavy-drinkers and poor sleepers. Lastly, future investigations should use measures relatively more robust to retrospective reporting error for drinking (e.g., Timeline Follow-Back; Sobell and Sobell 1992) and daily sleep behaviors (e.g., Consensus Sleep Diary; Carney et al. 2012), as well as objective sleep assessment (e.g., actigraphy).

## Conclusion

Despite mounting evidence that multiracial adolescents may be at greater risk than their monoracial peers for diverse adverse health outcomes, common underlying risk mechanisms remain under-researched. This cross-sectional study fills a gap in the literature by investigating multiracial relative to monoracial adolescents (rather than monoracial comparisons) and evaluating a theoretically-indicated, multifinal risk pathway to sleep and drinking disparities among adolescents. The results highlight a serial indirect risk pathway involving discrimination experiences and in turn negative mood underlying associations of multiracial status with sleep problems, but not drinking frequency (although this risk pathway was significant after excluding White students from analysis). Although longitudinal replication is needed, current preliminary findings may guide the development of prevention and/or intervention programming to mitigate adverse health effects of discrimination experiences.

## Figures and Tables

**Table 1:** Race combinations for full sample and prevalence of Hispanic/Latinx ethnicity

Race	Frequency (%)	Hispanic/Latinx Ethnicity (%)
**Monoracial**	**342 (83%)**	**24 (6%)**
Black only	171 (41%)	12 (3%)
White only	89 (22%)	4 (1%)
Asian only	74 (18%)	2 (<1%)
Other only^a^	10 (2%)	6 (1%)
**Multiracial**	**70 (17%)**	**27** (7%)
Black and White	14 (3%)	3 (1%)
Black and Asian	1 (<1%)	0 (0%)
Black and Other	17 (4%)	8 (2%)
White and Asian	2 (1%)	0 (0%)
White and Other	2 (1%)	2 (<1%)
Asian and Other	2 (1%)	0 (0%)
≥ 3 Categories	20 (5%)	9 (2%)
Unspecified^b^	12 (3%)	5 (1%)
**Total**	**414 (100%)**	**51 (12%)**

Note.

a Other=American Indian or Alaska Native and Native Hawaiian or Other Pacific Islander.

b Unspecified multiracial students endorsed multiracial status but did not indicate specific racial groups.

**Table 2:** Descriptive Statistics and Bivariate Correlations Among Study Variables

			*Correlation Coefficients*											
Variable (possible range)	*N* (%)	*M (SD)* or %	1	2	3	4	5	6	7	8	9	10	11	12
1. Multiracial (1 vs. 0)	414 (100%)	17%	-											
2. Age	412 (99%)	16.00 (1.08)	−.03	-										
3. Male Sex (1 vs. 0)	414 (100%)	43%	−.08	**.11**	-									
4. Hispanic/Latinx Ethnicity (1 vs. 0)	414 (100%)	12%	**.36**	.05	−.04	-								
5. Racial/Ethnic Identity (1 – 5)	404 (98%)	3.31 (1.10)	.05	.05	−.08	−.03	-							
6. Eligibility for Free/Reduced-Price Lunch (1 vs. 0)	412 (99%)	87%	.06	.03	−.09	.07	.04	-						
7. Discrimination Experiences (0 – 31)	404 (98%)	9.67 (7.16)	**.14**	.05	−.02	.02	.08	.09	-					
8. Negative Mood (0 – 12)	402 (97%)	3.06 (3.16)	.04	.02	−**.17**	−.04	−.02	−.00	**.40**	−				
9. Insomnia Symptom Severity (0 – 21)	407 (98%)	8.18 (5.14)	.02	**.11**	−**.12**	−.03	−**.12**	.08	**.22**	**.38**	-			
10. Insufficient (<7hr) Weekday Sleep Duration (1 vs. 0)	407 (98%)	50%	.05	.07	−.04	.09	−.02	.05	**.10**	**.14**	**.20**	-		
11. Insufficient (<7hr) Weekend Sleep Duration (1 vs. 0)	407 (98%)	33%	−.00	.02	.06	.09	−.05	**.10**	.09	−.00	.03	**.21**	-	
12. Past-Year Drinking Frequency (0 – 6)	173 (42%)	1.34 (1.26)	−.10	**.18**	.05	−.07	−.15	−**.25**	−.03	.13	−.08	−.10	.10	-
13. Past-2-Week Binge Drinking Frequency (0 – 5)	166 (40%)	0.30 (0.89)	.11	.01	.15	.13	.02	.03	.01	.06	.09	−.10	.11	.13

*Note*. Pearson’s correlation coefficients are reported for two continuous variables; Spearman’s coefficients (*r*_*s*_) are reported for continuous and dichotomous variables; Phi coefficients (*r*_*φ*_) are reported for two dichotomous variables. Significant coefficients at *p*<.05 are highlighted in bold font.

**Table 3:** Baseline Demographic and Sleep Characteristics and Group Comparisons

	Race					Comparison Statistic	Post Hoc Comparison
Variable (possible range)	Multiracial *n*=70 (17%)	Black *n*=171 (41%)	White *n*=89 (22%)	Asian *n*=74 (18%)	Other *n*=10 (2%)		
Age	15.94 (1.10)	16.10 (1.04)	15.74 (0.88)	16.14 (1.27)	15.96 (1.26)	F(4,407)=2.10	
Male Sex (1 vs. 0)	34%	43%	49%	46%	20%	X2(4)=6.09	
Hispanic/Latinx Ethnicity (1 vs. 0)	39%	7%	5%	3%	60%	X2(4)=81.35***	M,O>B,W,A***
Racial/Ethnic Identity (1 – 5)	3.48 (1.01)	3.26 (1.18)	3.08 (1.10)	3.59 (0.94)	2.98 (1.01)	F(4,399)=2.82*	A>W*
Eligibility for Free/Reduced-Price Lunch (1 vs. 0)	91%	92%	74%	85%	100%	X2(4)=20.81***	M>W*; B>W***
Discrimination Experiences (0 – 31)	11.97 (7.58)	9.17 (7.29)	8.60 (6.19)	9.42 (7.22)	13.70 (6.43)	F(4,399)=3.31*	M>W*
Negative Mood (0 – 12)	3.45 (3.48)	2.54 (3.04)	3.75 (3.22)	3.06 (3.03)	3.40 (2.32)	F(4,397)=2.48*	W>B*
Insomnia Symptom Severity (0 – 21)	8.31 (5.33)	8.04 (5.03)	8.05 (5.05)	8.32 (5.14)	9.90 (6.95)	F(4,402)=0.35	
Insufficient (<7hr) Weekday Sleep Duration (1 vs. 0)	56%	51%	52%	44%	60%	X2(4)=2.53	
Insufficient (<7hr) Weekend Sleep Duration (1 vs. 0)	34%	41%	28%	21%	70%	%2(4)=16.01**	B,O>A*
Past-Year Drinking Frequency (0 – 6)	1.11 (1.14)	1.38 (1.24)	1.77 (1.37)	0.91 (1.11)	1.50 (1.29)	*F* (4,168)=2.77*	W>A*
Past-2-Week Binge Drinking Frequency (0 – 5)	0.60 (1.38)	0.28 (0.81)	0.09 (0.36)	0.22 (0.55)	1.00 (2.00)	F(4,161)=2.39	

*Note. N*=166–414. M=Multiracial; B=Black; W=White; A=Asian; O=other race (i.e., American Indian or Alaska Native and Native Hawaiian or Other Pacific Islander). Group comparisons and post-hoc analyses were conducted using one-way ANOVA tests for continuous variables and non-parametric Kruskal-Wallis tests for dichotomous variables, with pairwise comparisons adjusted by Bonferroni correction for multiple tests.

* *p* < .05.

** *p* < .01.

*** *p* < .001.
